# Ameliorative effect of water spinach, *Ipomea aquatica* (Convolvulaceae), against experimentally induced arsenic toxicity

**DOI:** 10.1186/s12967-015-0430-3

**Published:** 2015-03-05

**Authors:** Tarun K Dua, Saikat Dewanjee, Moumita Gangopadhyay, Ritu Khanra, Muhammad Zia-Ul-Haq, Vincenzo De Feo

**Affiliations:** Advanced Pharmacognosy Research Laboratory, Department of Pharmaceutical Technology, Jadavpur University, Kolkata, 700032 India; Biophysics Division, Saha Institute of Nuclear Physics, Kolkata, 700064 India; The Patent Office, Karachi, 74470 Pakistan; Department of Pharmacy, University of Salerno, Fisciano, Salerno, 84084 Italy

**Keywords:** Apoptosis, As toxicity, *Ipomea aquatica*, NaAsO_2_, Oxidative stress

## Abstract

**Background:**

*Ipomea aquatica* (Convolvulaceae) is traditionally used against Arsenic (As) poisoning in folk medicines in India. The present study was designed to explore the therapeutic role of aqueous extract of *I. aquatica* (AEIA) against As-intoxication.

**Methods:**

AEIA was chemically standardized by spectroscopic and chromatographic analysis. The cytoprotective role of AEIA was measured on isolated murine hepatocytes. The effect on redox status were measured after incubating the hepatocytes with NaAsO_2_ (10 μM) + AEIA (400 μg/ml). The protective effect of AEIA (400 μg/ml) in expressions of apoptotic proteins were estimated *in vitro*. The protective role of AEIA was measured by *in vivo* assay in mice. Haematological, biochemical, As bioaccumulation and histological parameters were evaluated to ensure the protective role of AEIA (100 mg/kg) against NaAsO_2_ (10 mg/kg) intoxication.

**Results:**

Phytochemical analysis revealed presence of substantial quantities of phenolics, flavonoids, saponins and ascorbic acid in AEIA. Incubation of murine hepatocytes with AEIA (0–400 μg/ml) + NaAsO_2_ (10 μM) exerted a concentration dependent cytoprotective effect. Incubation of murine hepatocytes with NaAsO_2_ (10 μM, ~ IC_50_) induced apoptosis via augmenting oxidative stress. NaAsO_2_ treated hepatocytes exhibited significantly (p < 0.01) enhanced levels of ROS production, lipid peroxidation and protein carbonylation with concomitant depletion of antioxidant enzymes (p < 0.05-0.01) and GSH (p < 0.01) levels. However, AEIA (400 μg/ml) + NaAsO_2_ (10 μM) could significantly (p < 0.05-0.01) reinstate the aforementioned parameters to near-normal status. Besides, AEIA (400 μg/ml) could significantly counteract (p <0.05-0.01) ROS mediated alteration in the expressions of apoptotic proteins viz. Bcl-2, BAD, Cyt C, Apaf 1, caspases, Fas and Bid. In *in vivo* bioassay, NaAsO_2_ (10 mg/kg) treatment in mice caused significantly (p < 0.05-0.01) elevated As bioaccumulation, ATP levels, DNA fragmentations and oxidative stress in the liver, kidney, heart, brain and testes along with alteration in cytoarchitecture of these organs. In addition, the serum biochemical and haematological parameters were significantly (p < 0.05-0.01) altered in the NaAsO_2_-treated animals. However, concurrent administration of AEIA (100 mg/ml) could significantly reinstate the NaAsO_2_-induced pathogenesis.

**Conclusion:**

Presence of substantial quantities of dietary antioxidants within AEIA would be responsible for overall protective effect.

**Electronic supplementary material:**

The online version of this article (doi:10.1186/s12967-015-0430-3) contains supplementary material, which is available to authorized users.

## Background

Arsenic (As), a harmful metalloid, is one of the oldest known poison. It is a ubiquitous element with a ranking of 20 in the earth’s crust and 14 in sea water [1]. Naturally occurring As compounds (arsenicals) principally exist in two forms: inorganic arsenic (in combination with oxygen, chlorine, sulfur etc.) and organic arsenic (in combination with carbon and hydrogen). However, inorganic arsenicals are considered to be the potential threat in arsenicosis. Trivalent inorganic arsenicals (arsenite) in underground water are major cause of arsenicosis affecting more than 140 million people in at least 70 countries like Afghanistan, Argentina, Bangladesh, Cambodia, Chile, China, India, Mexico, Mongolia, Myanmar, Nepal, Pakistan, Taiwan, Vietnam, sub-Shahran Africa and USA [2,3]. Following oral consumption of As contaminated drinking water, arsenites are absorbed through the gastrointestinal tract and distributed into various organs [4]. However, arsenicals could also enter into the body via inhalation and dermal absorption. Following bioaccumulation, As participates in cellular redox events leading to generation of excessive reactive oxygen species (ROS). Besides, As has strong affinity toward thiol group, which is proposed to another pathway of developing oxidative stress during arsenicosis [5]. The increasing oxidative stress and depletion of endogenous antioxidant system during As-intoxication trigger the apoptotic events. Oxidative stress also targets cellular macromolecules and causes oxidative damage of DNA, peroxidative damge of membrane lipids, and carbonylation of proteins and thereby participates in cellular pathophysiology [6]. As affects almost all principle organs and tissues like liver, kidneys, lungs, heart, testes, brain and blood. Despite As toxicity as a global problem, there is no reliable, specific and safe treatment of arsenicosis. The primary treatment of As intoxication remains the chelation therapy with synthetic chelating agents like 2,3-dimercaprol, meso-2,3-dimercaptosuccinic acid and 2,3-dimercaptopropane-1-sulfonate [3,7]. However, many adverse effects including removal of essential metals and redistribution of As within tissues largely limited their clinical values [3,7,8]. On other hand, dietary antioxidants are known for long timr for their effectiveness against oxidative stress related complications without producing notable toxic manifestations. Considering the correlation between As toxicity and oxidative stress, researchers are looking forward for utilizing the dietary antioxidant/s to combat arsenicosis. In this issue, the World Health Organization (WHO) also recommended the ingestion of β-carotene, ascorbic acid, tocopherol and/or trace elements like Zn and Se to reverse symptoms of arsenicosis [9].

**Figure 1:**
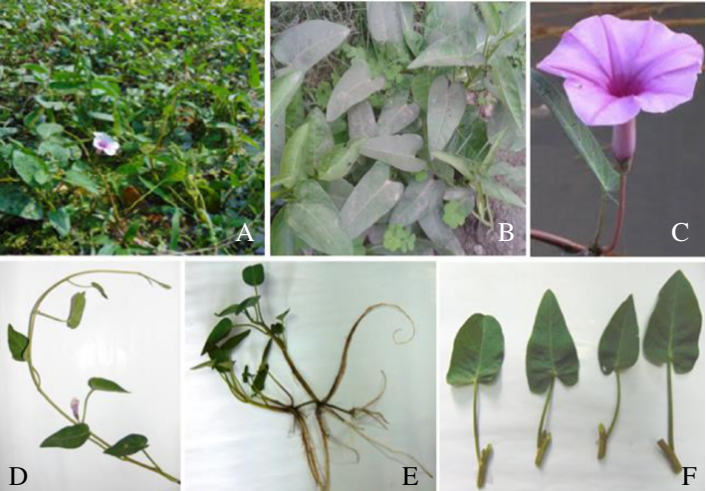
**The morphology of** ***I. aquatica*** **Forssk.** Panel **A.** The plant floating on water; Panel **B.** The plant creeping on moist soil; Panel **C.** The funnel form flower of *I. aquatica*; Panel **D.** flowering twig with simple and alternate leaves; Panel **E.** rooting at nodes; Panel **F.** leaves arise from nodes.

Ground water As contamination (greater than WHO permissible limit) is a major threat in Gangetic delta between India (West Bengal) and Bangladesh [1]. Nearly, 51 districts of these two Countries are seriously affected by severe arsenicosis. Water spinach, *Ipomea aquatica* Forssk. (Convolvulaceae), is a popular vegetable in this place. *I. aquatica* is an aquatic or semi-aquatic annual herb (Figure 1) creeping on moist soil/sand or floating on water. Stems are hollow, branched, and juicy up to 3 m long, to 1 cm in diameter. Leaves are simple, alternate, with glabrous petioles. The leaves are generally arrow headed but variable in shapes. Flowers are funnel form, solitary, with white to purple petals. Fruits are oval to spherical capsules becoming woody at maturity. The aerial parts of *I. aquatica* are consumed as green leafy vegetables due to their high nutritive values and consumed by the people of Southern Asia, India, Bangladesh and China. *I. aquatica* has been used in folk medicine against different diseases including diabetes [10], liver malfunction [11], constipation [12] and in the treatment of As poisoning [13]. Literature reviews revealed presence of flavonoids, phenolic compounds, β-catotene and ascorbic acid in *I. aquatica* [14]. Despite the ethnomedicinal literature revealed the effectiveness of *I. aquatica* against arsenicosis, the plant has not been yet explored scientifically to validate this folklore claim. Considering the ethnomedicinal values of *I. aquatica* against As poisoning, the current study was designed to evaluate the possible therapeutic role of aqueous extract of *I. aquatica* against As-intoxication. Since *I. aquatica* is a dietary vegetable, the edible (aqueous) extract was chosen in this study. Significant attempts were made to elucidate the mechanism of actions against arsenicosis and to establish the correlation between observed effects with the phytochemicals present within the test material.

## Materials and methods

### Chemicals

Bovine serum albumin (BSA), Dulbecco’s modified Eagle’s medium (DMEM), fetal bovine serum (FBS), Bradford reagent, Collagenase type I, antibodies for immunoblotting were purchased from Sigma-Aldrich Chemical Company (St. Louis, MO), USA. Solvents (HPLC grade) were obtained from Merck, Mumbai, India. Kits for measurement of serum biochemical parameters were purchased from Span diagnostic Ltd., India. 1-Chloro-2,4-dinitrobenzene, ammonium sulphate, sodium arsenite (NaAsO_2_), 2,4-dinitrophenylhydrazine, ethylene diaminetetraacetic acid, 5,5-dithiobis(2-nitrobenzoic acid), N-ethylmaleimide, nitro blue tetrazolium, reduced nicotinamide adenine dinucleotide, potassium dihydrogen phosphate, phenazinemethosulphate, sodium pyrophosphate, reduced glutathione, sodium azide, thiobarbituric acid, 5-thio-2-nitrobenzoic acid and trichloro acetic acid were purchased from Sisco Research Laboratory, Mumbai, India.

### Preparation of extract

Aerial parts of *I. aquatica* were collected in September, 2012 from West Bengal (22° 42′N, 87°32′E), India. The plant was authenticated by the Taxonomist, Botanical Survey of India, Shibpur, India and a voucher specimen (JU/PT/PC/07/12) has been deposited at the Advanced Pharmacognosy Research Laboratory, Department of Pharmaceutical Technology, Jadavpur University for future references. The plant material was dried under shade (<35°C, for 72 h), powdered and macerated with double distilled water containing 1% chloroform for 48 h (<35°C) with continuous stirring. The cellular debris were removed from extract by filtration and the resulting extract was lyophilized (Heto FD 3 Drywinner, USA) to get the crude extract AEIA (11.7%, w/w). The AEIA was suspended in tween 80 (1%) prior to each animal experiment, whereas for *in vitro* assay, AEIA was solubilized in DMSO (resultant ≤ 0.4% DMSO in contact to cells to avoid DMSO induced cytotoxicity).

### Phytochemical analysis

Quantitative analysis of phytoconstituents within AEIA was determined spectrophotometrically to evaluate total flavonoids [15], total phenolics [16], total saponins [17] and total carbohydrates [18]. Dionex Ultimate 3000 HPLC (hi pressure liquid chromatography) system (Dionex, Germany), equipped with a reverse phase (RP) C-18 column (250 × 4.6 mm, particle size 5 μ) and an UV detector was used for identification of individual phenolic and flavonoid compounds. Briefly, the standard markers and AEIA were dissolved in methanol (HPLC grade) and filtered by cellulose nylon membrane filter (0.45 μm). The aliquots of the filtrate were eluted with isocratic solvent mixtures comprising methanol:acetonitrile:acetic acid:o-phosphoric acid:water (20:10:1:1:20) for flavonoids, methanol:water:acetic acid (75: 24:1) for phenolics and phosphate buffer (pH 3.0; 25 mM) for ascorbic acid with a flow rate of 1 ml/min and detected at 352, 254 and 230 nm, respectively. Ascorbic acid was quantified by RP-HPLC analysis.

### Animals

Healthy male Swiss albino mice (25 ± 5 g) were obtained from the Ghosh Enterprise, Kolkata, India. The animals were kept in standard polypropylene cages and were fed standard diet and water *ad libitum*. Animals were kept under standard laboratory conditions of temperature (22 ± 2°C), humidity (40 ± 10%) and 12 h light–dark cycle. The principles of Laboratory Animals care [19] and the instructions given by our institutional animal ethical committee (Reg. no. 0367/01/C/CPCSEA) were followed throughout the experiment. The animals were acclimatized for a fortnight before starting experiment.

### *In vitro* bioassay

#### Hepatocytes isolation

Hepatocytes were isolated following the protocol of Sarkar and Sil [20] with little modification. Briefly, livers were separated aseptically from mice after CO_2_ euthanasia. The excised livers were washed with ice-cold PBS (pH 7.4) and hepatocytes were isolated by collagenase perfusion followed by mechanical disruption. Hepatocytes were passed through a wide bore syringe, filtered and centrifuged (500 rpm) for 5 min. The pellet was re-suspended in DMEM containing 10% FBS and incubated at 37°C and 5% CO_2_ tension.

### Determination of cytotoxic effect of NaAsO_2_

Concentration dependent cytotoxic effect of NaAsO_2_ was determined by cell viability assay using MTT (3-(4, 5-dimethylthiazolyl-2)-2, 5-diphenyltetrazolium bromide) [21]. Brifely, ~2 × 10^5^ cells/well were seeded in tissue culture plates and incubated at 37°C and 5% CO_2_ tension. After seeding of 24 h, the cells were treated with NaAsO_2_ (0.5-10000 μM). After 2 h, the cells were treated with 40 μl of FBS-DMEM medium containing MTT (2 mg/ml) and incubated for 4 h. After 4 h of incubation period, the cells were treated with DMSO (150 μl), and readings were noted spectrophotometrically at 570 and 630 nm. The differences between these two absorbances and a percentage of the corresponding controls were used to express cell viability. The experiment was repeated thrice.

#### Determination of cytoprotective activity of AEIA

Time and dose dependent cytoprotective role of AEIA was measured by cell viability assessment. Briefly, ~2 × 10^5^ cells/well were seeded in tissue culture plate and incubated at 37°C and 5% CO_2_. Twenty four h after seeding, the cells were incubated with NaAsO_2_ (10 μM) along with different doses of AEIA (0–400 μg/ml). The cell viabilities at different times (0.5-4 h) were determined by MTT assay [21]. Three independent assays were carried out.

#### Hoechst staining

Hoechst staining was used to detect viable nuclei by evaluation of nuclear count and morphologies under fluorescence microscopy [22]. Briefly, 2000 cells/well were seeded in tissue culture plates and incubated at 37°C and 5% CO_2_. Twenty four h after seeding, the cells were incubated with NaAsO_2_ (10 μM) + AEIA (400 μg/ml). After 2 h, the media was removed and the cells were washed with PBS. Then the cells were fixed in paraformaldehyde (4%) in PBS for 20 min. After 20 h, paraformaldehyde was removed and the cells were washed with PBS. The cells were incubated with Hoechst 33258 (5 μg/ml in PBS) for 20 min. Fluorescent nuclei were scored and categorized according to the condensation and staining characteristics of chromatin.

#### Assay of antioxidant markers

Different sets of hepatocytes, each containing 1 ml of suspension (~2 × 10^6^ cells/ml) were used in experiments. The prophylactic role of AEIA against NaAsO_2_-intoxication was analyzed by incubating hepatocytes with extracts (400 μg/ml) and NaAsO_2_ (10 μM) together for 2 h at 37°C. One set of hepatocytes incubated with NaAsO_2_ (10 μM) and one set without any treatment were kept as toxic and normal control, respectively. Generation of intracellular ROS has been quantified by the slightly modified method of LeBel and Bondy [23] and 2′, 7′-dichlorofluorescein (DCF) formation was measured by using a fluorescence microscopy. The degree of lipid peroxidation was measured by estimating thiobarbituric acid reactive substances (TBARS) following the protocol of Ohkawa et al. [24]. The level of protein carbonylation was determined in accordance to Uchida and Stadtman [25]. A previously reported method of Ghosh et al. [26] was used to assess the level of intracellular antioxidant enzymes like catalase (CAT), superoxide dismutase (SOD), glutathione peroxidase (GPx), glutathione reductase (GR) and glutathione-S-transferase (GST), while, non-enzymatic antioxidant viz. reduced glutathione (GSH) level was measured by the method of Hissin and Hilf [27].

#### Immunoblotting of signaling proteins

Cells under different treatments were separated by centrifugation (800 g, for 5 min). The isolated cells were treated with 30 μl of lysis buffer and incubated within ice for 30 min followed by centrifugation (14000 g, for 10 min) to remove cellular debris and to isolate proteins as supernatant. Samples containing proteins (20 μg) were subjected to SDS-PAGE (10%) and transferred to a nitrocellulose membrane following standard dry transfer protocol. Membranes were blocked (4°C; 1 h) using Tris-buffered saline with 0.1% Tween 20 (TBST) containing non-fat dry milk (5%) to prevent non-specific binding with gentle shaking. The membranes were washed with TBST (pH 7.6) for three times (5 min, each) with gentle shaking. Then the membranes were incubated with primary antibodies viz. anti-caspase 3 and 9 (1:2000), anti-caspase 8 (1:1000), anti-Bad (1:3000), anti-Bcl-2 (1:3000), anti-Fas and anti-Bid (1: 1000), anti-cytochrome (Cyt) C and anti-Apaf (apoptotic protease activating factor) 1 (1: 2000) in TBST containing 5% of BSA at 4°C overnight with gentle shaking. The membranes were washed with TBST (pH 7.6) for three times (5 min, each) with gentle shaking and incubated with appropriate HRP conjugated secondary antibody (1:3000 dilution) in TBST containing non-fat dry milk (5%) for 1 h at room temperature and finally developed by the HRP substrate 3, 3′-diaminobenzidine tetrahydrochloride system (Bangalore Genei, India). The Western blot analysis and densitometry studies were performed using Image Lab 5.2 software (Bio-Rad, CA, USA).

### *In vivo* bioassay

#### Experimental design

Eighteen Swiss albino mice (♂) were divided into three groups (n = 6) and they were treated as follows:

- Group I: Normal control, animals received only 1% tween 80 (p.o., once daily) for 15 days;
- Group II: Toxic control, mice treated with NaAsO_2_ (10 mg/kg body weight, p.o., once daily) for 10 days
- Group III: Animals were treated with AEIA (100 mg/kg body weight, p.o., once daily) for 15 days, prior to NaAsO_2_ (10 mg/kg body weight, p.o., once daily) for 10 days [28].

Based on *in vitro* data and previous observations, an optimum dose of AEIA of 100 mg/kg body weight was used *in vivo* study as per the recommendation of Institutional animal ethical committee.

On day 16, the treated mice were subjected to CO_2_ euthanasia and sacrificed by cervical dislocation. For haematological assays, blood samples were collected from retro-orbital venous plexus in Eppendorf tubes rinsed with anticoagulant before sacrificing. The organs (liver, kidney, brain, heart and testes) were excised, cleaned and washed three times with PBS to remove adherent blood. The organs were homogenized in 0.1 M Tris–HCl-0.001 M EDTA buffer (pH 7.4) and centrifuged (12,000 g; 30 min) at 4°C. The supernatant were collected and used for assaying biochemical parameters.

#### Haematological and serum biochemical parameters

Total erythrocyte count and haemoglobin content were determined by using standard laboratory procedures. Serum biochemical parameters viz. alanine aminotransferase (ALT), aspartate aminotransferase (AST), creatinine kinase (CK), cholesterol, lactate dehydrogenase (LDH), urea, and triglycerides were estimated by commercially available kits (Span Diagnostic Limited, India).

#### Assessment of antioxidant markers related to organ dysfunction

Distribution of As in tissues was measured by flame atomic absorption spectroscopy [29]. The antioxidant enzymes, intercellular ROS, lipid peroxidation, protein carbonylation, and non-enzymatic antioxidant were assayed following standard assay protocols mentioned earlier. Co-enzymes Q (Q_9_ and Q_10_) were isolated and estimated by HPLC a previously described method of Zhang et al. [30]. Intracellular ATP concentrations were assessed by commercial kits (Abcam, Cambridge, USA) following manufacturer protocol.

#### Histological studies

The mice organs’ from different groups were fixed in buffered formalin (10%) and were processed for paraffin sectioning. Organ sections (5 μm) were stained with hematoxylin and eosin to study the histology of organs [31].

### Statistical analysis

Data were statistically analyzed by one way ANOVA and represented as mean ± SE followed by Dunnett’s *t*-test using GRAPHPAD INSTAT version 3.05 (GraphPad software, CA, U.S.A). The values were considered significant when *p* < 0.05. The graphs were drawn using GRAPHPAD PRISM version 6 (GraphPad software, CA, U.S.A).

## Results

### Phytochemical analysis

Quantitative phytochemical analysis revealed substantial quantities of flavonoids (~37.9 mg/g^DW^), phenolics (~22.7 mg/g^DW^), saponins (~51.2 mg/g^DW^), carbohydrates (~132.7 mg/g^DW^) and ascorbic acid (~3.1 mg/g^DW^). The identification of phenolics and flavonoids were done by comparing retention time (R_t_) and UV spectra with standard flavonoids and phenolic compounds by using RP-HPLC analysis. The results revealed presence of flavonoids namely myricetin, quercetin and apigenin in AEIA (Additional file 1: Figure S1, Panel A). In search of phenolics, AEIA revealed presence of gallic acid and chlorogenic acid (Additional file 1: Figure 1, Panel B). HPLC analysis of ascorbic acid has been depicted in Additional file 1: Figure S1, Panel C.

### Effect of AEIA against NaAsO_2_-intoxication *in vitro*

#### Dose dependent effect of NaAsO_2_ induced cytotoxicity

**Figure 2:**
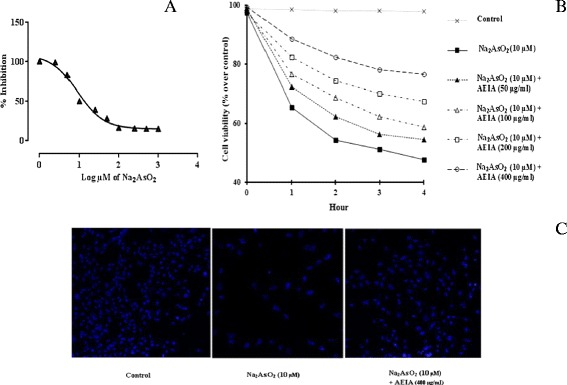
**Cell viability studies in absence (NaAsO**_**2**_ **) and presence of AEIA (NaAsO**_**2**_ **+ AEIA)** ***in vitro.*** Panel **A.** Effect of NaAsO_2_ at different concentrations in cell viability in isolated mouse hepatocytes. Panel **B.** Time and dose-dependent effect on cell viability in absence (NaAsO_2_) and presence of AEIA (NaAsO_2_ + AEIA) in isolated murine hepatocytes. Values were expressed as mean ± SE (n = 3). Panel **C.** Hoechst staining of murine hepatocytes in absence (NaAsO_2_) and presence of AEIA (NaAsO_2_ + AEIA).

Cell viability is an important indicator to find the degree of cytotoxicity. Figure 2A depicts the dose dependent effect of NaAsO_2_ in isolated murine hepatocytes. Hepatocytes incubated with NaAsO_2_ (0.5-10000 μM) for 2 h exhibited the reduction in cell viability in a concentration dependant manner. The IC_50_ value was found to be 9.8 μM (~10 μM). Based on observed IC_50_ value, all the subsequent *in vitro* experiments employed NaAsO_2_ (10 μM) as a toxic control and to evaluate prophylactic role of AEIA.

#### Dose dependent cytoprotective role of AEIA against NaAsO_2_ induced cytotoxicity

Results of the dose dependent protective role of AEIA (50–400 μg/ml) in NaAsO_2_ (10 μM) induced cytotoxicity in hepatocytes have been depicted in Figure 2B. NaAsO_2_ (10 μM, ~ IC_50_) exposure caused significant reduction in cell viability up to 4 h. Simultaneous treatment of hepatocytes with AEIA (50–400 μg/ml) and NaAsO_2_ (10 μM) prevented the reduction in cell viability in a concentration dependant manner. However, after 2 h the cytoprotective effect appeared to be optimum. Based on this observation, the concentration of AEIA, and incubation time have been calibrated as 400 μg/ml and 2 h, respectively, for subsequent *in vitro* study.

#### Effect on Hoechst staining

The anti-cytotoxic effect of AEIA was estimated by fluorescence microcopy after Hoechst staining (Figure 2C) of hepatocytes under different treatments. NaAsO_2_ (10 μM) treated hepatocytes showed significantly less number of visible nuclei; however, the visible nuclei exhibited specific pattern of morphological changes, condensation, fragmentation of the nuclei and chromatin condensation. Simultaneous incubation of hepatocytes with AEIA (400 μg/ml) and NaAsO_2_ (10 μM) could significantly counteract the cytotoxic effect of NaAsO_2_ (10 μM) and restore nuclear morphology near to normalcy.

#### Effect on redox status

**Figure 3:**
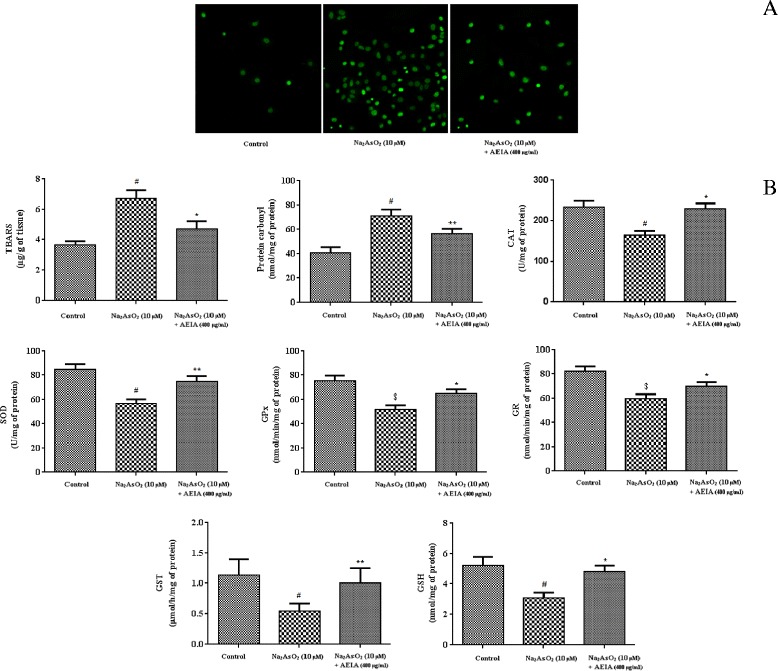
**The effect on cellular redox markers in absence (NaAsO**_**2**_ **) and presence of AEIA (NaAsO**_**2**_ **+ AEIA) in isolated murine hepatocytes.** Panel **A.** Effect on intracellular ROS production was detected by fluorescence microscopy using DCF-DA in Cd-exposed hepatocytes in absence (NaAsO_2_) and presence of AEIA (NaAsO_2_ + AEIA). Panel **B.** Effect on antioxidant parameters viz. lipid peroxidation, protein carbonylation, CAT, SOD, GR, GPx, GST and GSH in absence (NaAsO_2_) and presence of AEIA (NaAsO_2_ + AEIA) in isolated murine hepatocytes. Values were expressed as mean ± SE (n = 3). ^#^Values differed significantly from normal control (p < 0.01). ^$^ Values differ significantly from normal control (p < 0.05). *Values differed significantly from NaAsO_2_ control (p < 0.05). **Values differed significantly from NaAsO_2_ control (p < 0.01). CAT unit, ‘U’ is defined as μmoles of H_2_O_2_ consumed per minute. SOD unit, ‘U’ is defined as the μmoles inhibition of NBT reduction per minute.

Figure 3 represented the effect of AEIA against NaAsO_2_ induced alteration of antioxidant markers in isolated in murine hepatocytes. Intracellular ROS production plays a principle role in NaAsO_2_ induced oxidative stress. Production of intracellular ROS was measured by fluorescence microscopy using fluorescent dye DCF (Figure 3A). It has been observed that NaAsO_2_ (10 μM) exposure led to an increased production of intracellular ROS, which could be prevented by simultaneous incubation of NaAsO_2_ (10 μM) along with AEIA (400 μg/ml). The extents of lipid peroxidation (TBARS level) and protein carbonylation were significantly (p < 0.01) increased in the hepatocytes incubated with NaAsO_2_ (10 μM) when compared with control hepatocytes (Figure 3B). Simultaneous incubation of AEIA (400 μg/ml) and NaAsO_2_ (10 μM) could significantly inhibit lipid peroxidation (p < 0.05) and protein carbonylation (p < 0.01) when compared to NaAsO_2_ (10 μM) treated group. Cellular antioxidant enzymes protect biological macromolecules from oxidative damage. In this study, the levels of CAT, SOD, GPx, GR and GST were significantly (p < 0.05-0.01) reduced in NaAsO_2_ treated hepatocytes (Figure 3B). However, concurrent incubation of hepatocytes with AEIA (400 μg/ml) and NaAsO_2_ (10 μM) could significantly (p < 0.05-0.01) revert the levels of antioxidant enzymes near to normalcy.

#### Effect on intrinsic and extrinsic pathways of cell death

**Figure 4:**
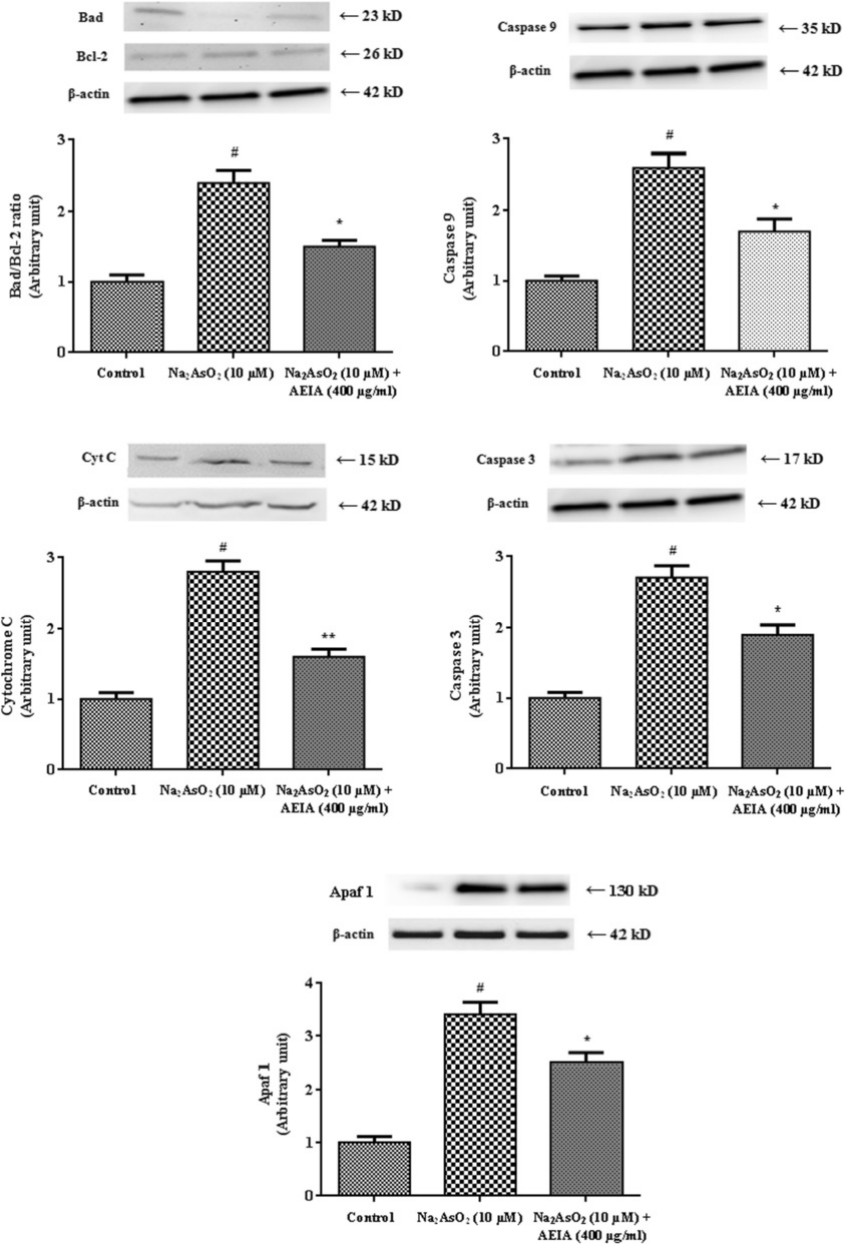
**Respective western blot analysis of Bad, Bcl-2, cyt-C, caspase 9, caspase 3 and Apaf 1 in absence (NaAsO**_**2**_ **) and presence of AEIA (NaAsO**_**2**_ **+ AEIA) in isolated murine hepatocytes.** The relative band intensities were measured and the normal control band was given an arbitrary value of 1. β-actin was used as a loading protein. Values were expressed as mean ± SE (n = 3). ^#^Values differed significantly (p < 0.01) from normal control. *Values differed significantly (p < 0.05) from NaAsO_2_ control. **Values differed significantly (p < 0.01) from NaAsO_2_ control.

Involvement of apoptosis indicates cellular damage under redox challenged cellular atmosphere. Apoptosis is synchronized by complex interactions between anti- and pro- apoptotic proteins followed by activation of caspases. In this study, the involvements of intrinsic and extrinsic pathways have been evaluated by western blot analysis. The immunoblot analysis of intrinsic transcription proteins have been depicted in Figure 4. The experimental observations suggested that NaAsO_2_ (10 μM) significantly up-regulated the pro-apoptotic (Bad) and down-regulated the anti-apoptotic (Bcl-2) proteins, which caused a significant (p < 0.01) increase in Bad/Bcl-2 ratio in isolated murine hepatocytes. Significantly increased (p < 0.01) expression of cytosolic Cyt C in association with activation (p < 0.01) of caspase 9 and 3 demonstrated the involvement of intrinsic apoptotic pathway in pathogenesis in hepatocytes incubated with NaAsO_2_ (10 μM). In present study, immunoblot analysis of Apaf 1 showed that NaAsO_2_ exposure caused a significant (p < 0.01) increase in the expression of Apaf 1. AEIA (400 μg/ml) co-treatment, however, could significantly inhibit NaAsO_2_-induced up-regulation of Cyt C (p < 0.01), caspase 9 (p < 0.05), caspase 3 (p < 0.05) and Apaf 1 (p < 0.05) along with reciprocate the regulation (p < 0.05) of Bad and Bcl-2.

**Figure 5:**
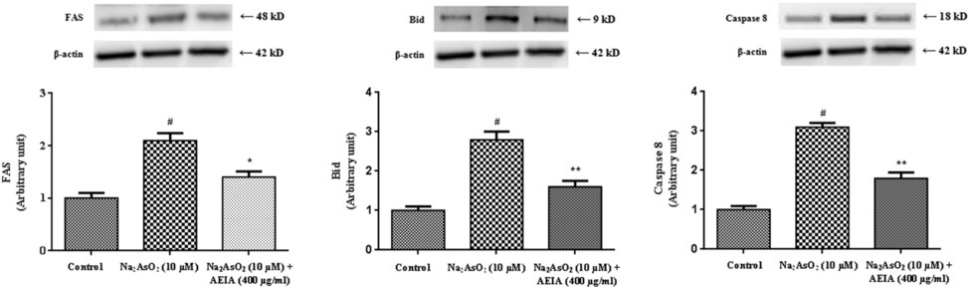
**Respective western blot analysis of Fas, Bid and caspase 8 in absence (NaAsO**_**2**_ **) and presence of AEIA (NaAsO**_**2**_ **+ AEIA) in isolated murine hepatocytes**. The relative band intensities were measured and the normal control band was given an arbitrary value of 1. β-actin was used as a loading protein. Values were expressed as mean ± SE (n = 3). ^#^Values differed significantly (p < 0.01) from normal control. *Values differed significantly (p < 0.05) from NaAsO_2_ control. **Values differed significantly (p < 0.01) from NaAsO_2_ control.

To study the effect of mitochondria independent apoptosis (extrinsic pathway), immunoblot analysis of Bid, caspase-8 and FAS were performed (Figure 5). NaAsO_2_ (10 μM) caused significant (p < 0.01) up-regulation of Bid, FAS and caspase 8 in isolated murine hepatocytes, which indicated the involvement of extrinsic pathway of apoptosis, simultaneously. However, concurrent treatment of the cells with AEIA (400 μg/ml) could significantly (p < 0.05-0.01) prevent the As-mediated up-regulation of transcription levels of extrinsic apoptotic proteins.

### Effect of AEIA against NaAsO_2_-intoxication *in vivo*

#### Effect on haematological and serum biochemical parameters

Haematological and serum biochemical parameters serve as first impression of physiological abnormalities and/or incidence of toxic manifestations. The effects of different treatments on haematological and serum biochemical parameters of experimental mice were shown in Table 1.

**Table 1:** **Effect on haematological and serum biochemical parameters in absence (NaAsO**_**2**_ **) and presence of AEIA (AEIA + NaAsO**_**2**_ **) in mouse**

**Groups**	**Parameters**								
	**Total erythrocyte count (x10** ^**6/**^ **mm** ^**3**^ **)**	**Haemoglobin (g/dl)**	**ALT (IU/l)**	**AST (IU/l)**	**Urea (mg/dl)**	**Lactate dehydrogenase (U/l)**	**Creatinine kinase (IU/ mg protein)**	**Cholesterol (mg/dl)**	**Triglycerides (mg/dl)**
I	6.05 ± 0.28	9.12 ± 0.79	72.21 ± 4.11	53.33 ± 3.44	24.17 ± 2.25	32.48 ± 2.11	205.56 ± 12.33	132.45 ± 7.11	122.41 ± 7.12
II	3.25 ± 0.22^#^	5.24 ± 0.76^#^	154.22 ± 7.43^#^	78.35 ± 4.72^#^	58.11 ± 3.41^#^	64.04 ± 3.50^#^	284.67 ± 16.17^$^	191.14 ± 7.22^#^	238.33 ± 10.94^#^
III	4.98 ± 0.27^**^	8.17 ± 0.54^**^	105.23 ± 5.02^*^	59.67 ± 3.92^**^	42.14 ± 2.01^**^	54.92 ± 3.88*	231.71 ± 17.92^*^	161.45 ± 6.25^*^	168.85 ± 9.15^**^

Values were expressed as mean ± SE (n = 6). ^#^Values differ significantly from normal control (p < 0.01). ^$^Values differ significantly from normal control (p < 0.01). ^*^Values differ significantly from NaAsO_2_ control (p < 0.05). ^**^Values differ significantly from NaAsO_2_ control (p < 0.01). Group I: Normal control; Group II: NaAsO_2_ (10 μM) control; Group III: NaAsO_2_ (10 μM) + AEIA (400 μg/ml).

The haematological parameters exhibited significant (p < 0.01) reduction in total erythrocyte counts and haemoglobin content in NaAsO_2_ (10 mg/kg) treated mice (group II). However, simultaneous administration of AEIA (100 mg/kg) along with NaAsO_2_ (10 mg/kg) could significantly reinstate the erythrocyte counts (p < 0.01) and haemoglobin content (p < 0.01) to near-normal status. NaAsO_2_ (10 mg/kg) treated mice exhibited a significant (p < 0.01) increase in AST, ALT, urea, lactate dehydrogenase, creatinine kinase, cholesterol and triglyceride levels. However, concurrent administration of AEIA (100 mg/kg) along with NaAsO_2_ (10 mg/kg) could significantly reinstate the serum biochemical parameters near to normalcy.

#### Effect on ss bioaccumulation

**Figure 6:**
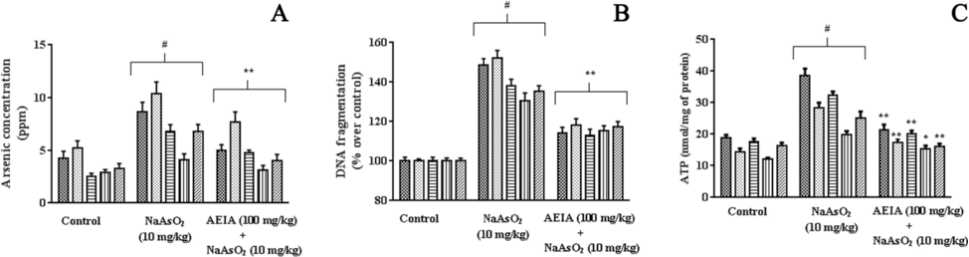
**Effect on As-bioaccumulation (A), DNA fragmentation (B) and ATP (C) in absence (NaAsO**_**2**_ **) and presence of AEIA (NaAsO**_**2**_ **+ AEIA) in liver, kidney, heart, brain and testes in experimental mice.** Values were expressed as mean ± SE (n = 6). ^#^Values differed significantly (p < 0.01) from normal control. *Values differed significantly (p < 0.05) from NaAsO_2_ control. **Values differed significantly (p < 0.01) from NaAsO_2_ control.

Accumulation of As in the critical organs is the major cause of As-pathogenesis. Therefore, the accumulation of As in tested organs under different treatments has been evaluated (Figure 6A). Exposure to NaAsO_2_ in mice increased significantly (p < 0.01) the bioaccumulation of As in kidney, liver, heart, brain and testes, as compared to normal mice. Order of intercellular As accumulation was kidney > liver > heart > testes > brain. Concurrent treatment of AEIA (100 mg/kg) along with NaAsO_2_ (10 mg/kg), however, could significantly (p < 0.01) attenuate intracellular As burden in aforementioned tissues, as compared to NaAsO_2_-intoxicated mice.

#### Effect on DNA fragmentation and ATP level

The percentages of DNA fragmentation in brain, kidney, heart, liver and testes of the experimental mice have been depicted in Figure 6B. Daily administration of NaAsO_2_ (10 mg/kg, up to 10 days) significantly increased (p < 0.01) the DNA fragmentation in the hepatic, renal, cardiac, cerebral and testicular tissues amounting ~ 148%, 152%, 138% and 130 and 135%, respectively. However, concurrent administration of AEIA (100 mg/kg) along with NaAsO_2_ (10 mg/kg) could significantly (p < 0.01) inhibit DNA fragmentation in aforementioned tissues. Intercellular ATP level within the tissues is an indicative of pathophysiological events. In this study, intracellular ATP level in the selected tissues was investigated (Figure 6C). It was observed that, NaAsO_2_ caused significant (p < 0.01) increment in intracellular ATP levels in mice tissues. Concurrent administration of AEIA (100 mg/kg) along with NaAsO_2_ (10 mg/kg) could significantly (p < 0.01) reinstate the ATP level near to normalcy.

#### Effect on redox status

**Figure 7:**
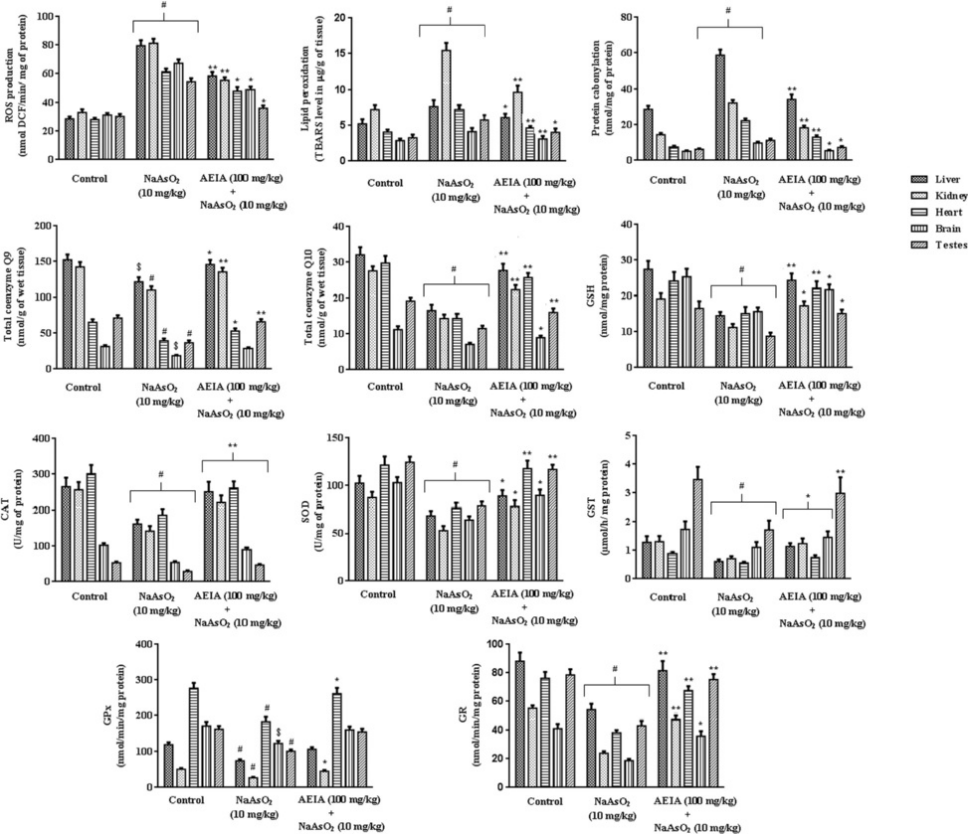
**Effect on antioxidant parameters viz. ROS production, lipid peroxidation, protein carbonylation, co-enzyme Q9, co-enzyme Q10, CAT, SOD, GR, GPx, GST and GSH in absence (NaAsO**_**2**_ **) and presence of AEIA (NaAsO**_**2**_ **+ AEIA) in experimental mice.** Values were expressed as mean ± SE (n = 6). ^#^Values differed significantly from normal control (p < 0.01). ^$^ Values differed significantly from normal control (p < 0.05). *Values differed significantly from NaAsO_2_ control (p < 0.05). **Values differed significantly from NaAsO_2_ control (p < 0.01). CAT unit, ‘U’ is defined as μmoles of H_2_O_2_ consumed per minute. SOD unit, ‘U’ is defined as the μmoles inhibition of NBT reduction per minute.

The effects of different treatments on redox status within selected organs of experimental mice have been depicted in Figure 7. In this study, As-intoxication significantly enhanced (p < 0.01) intercellular ROS production in liver, kidney, heart, brain and testes of experimental mice. Concurrent treatment of AEIA (100 mg/kg) along with NaAsO_2_ (10 mg/kg) could significantly (p < 0.05-0.01) prevent the As-mediated ROS generation within the selected tissues. Increased ROS production simultaneously enhanced lipid peroxidation and protein carbonylation. In this study, the extents of lipid peroxidation and protein carbonylation within the tissues of experimental mice were significantly (p < 0.01) increased in NaAsO_2_ (10 mg/kg) treated mice. However, concurrent treatment of AEIA (100 mg/kg) along with NaAsO_2_ (10 mg/kg) could significantly (p < 0.05-0.01) reinstate the ROS mediated augmented of lipid peroxidation and protein carbonylation in the tissues of experimental mice. Cellular ubiquinols (co-enzymes Q9 and Q10) participate in cellular electron carriers distributed in intracellular major organelles. In this study, a significant reduction of Q9 (p < 0.05-0.01) and Q10 (p < 0.01) levels in liver, kidney, heart, brain and testes of NaAsO_2_ (10 mg/kg) treated experimental mice suggested As-induced oxidative stress. However, simultaneous administration of AEIA (100 mg/kg) along with NaAsO_2_ (10 mg/kg) could significantly (p < 0.05-0.01) prevent As-induced reduction of ubiquinol levels in the selected tissues of experimental mice. Antioxidant enzymes and reduced glutathione (GSH) partake in cellular defense mechanism during oxidative stress. In this study, NaAsO_2_ (10 mg/kg) intoxication could significantly (p < 0.05-0.01) decrease the levels of antioxidant enzymes and GSH in the selected tissues of experimental mice, when compared with control group. Simultaneous administration of AEIA (100 mg/kg) along with NaAsO_2_ (10 mg/kg), however, could significantly (p < 0.05-0.01) prevent As-induced reduction of antioxidant enzymes and GSH levels to near-normal status in hepatic, renal, cardiac, cerebral and testicular tissues of experimental mice, when compared with NaAsO_2_ (10 mg/kg) control group.

#### Effect on histology of organs

**Figure 8:**
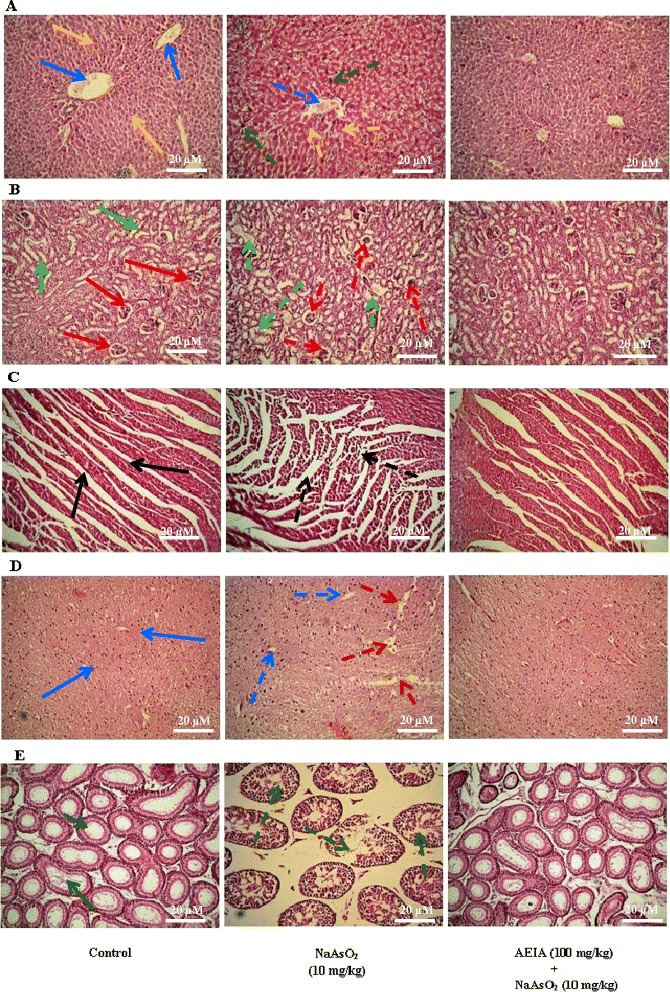
**Histological sections of different tested organs of experimental mice in absence (NaAsO**_**2**_ **) and presence of AEIA (NaAsO**_**2**_ **+ AEIA).** Untreated mice were kept as control to compare the structural changes caused by NaAsO_2_. Panel **A.** Histogram of liver sections; blue and yellow arrows represent normal portal vein and hepatocytes, respectively; dotted arrows represent the NaAsO_2_ mediated structural changes of portal vein (blue) and hepatocytes (yellow) with infiltrating leukocytes (green). Panel **B.** Histogram of kidney sections; red and green arrows represent normal glomerulus structure and renal tubules, respectively; dotted arrows represent the NaAsO_2_ mediated glomerular hypercellularity (red) and cloudy swelling of renal tubules (green). Panel **C.** Histogram of heart sections; black arrows indicated normal radiating pattern of cardiac muscle; black dotted arrows showed extensive degeneration in cardiac muscle during As-intoxication. Panel **D.** Histogram of brain sections; blue arrows represent normal normal cyto-architecture of brain; dotted arrows represent the NaAsO_2_ mediated development of vacuolated area of degenerated tissues (red) and diffused edema (blue). Panel **E.** Histogram of testes sections; green arrows represent normal seminiferous tubules; green dotted arrows represent NaAsO_2_ mediated marked degeneration in the seminiferous tubules in testes section.

The histological assessments of different organs viz. liver, kidney, heart, brain and testes have been presented in Figure 8. Histological assessments of liver of NaAsO_2_ (4 mg/kg) treated mice exhibited hepatocytes focal apoptosis with disrupted portal vein, when compared with normal liver section (Figure 8, panel: A). The histological examination of the renal tissues of the NaAsO_2_-control mice showed glomerular damage and cloudy swelling of tubules when compared with normal control (Figure 8, panel: B). As-intoxication led to extensive degeneration of cardiac muscle and interstitial fibrosis leading to abnormal radiation pattern of cardiac muscle (Figure 8, panel: C). The histological examination of brains of NaAsO_2_-control mice exhibited vacuolated area degenerated tissues and diffused edema as compared to normal control mice (Figure 8, panel: D). To study the histology of testes, severe cellular damage and degeneration of seminiferous tubules was observed in the testes from As-intoxicated mice (Figure 8, panel: E). However, treatment with AEIA (100 mg/kg) along with NaAsO_2_ (4 mg/kg) could reinstate the histology of selected organs near to normalcy.

## Discussion

As, a toxic element, exerts toxic manifestations in different organs. Generation of excessive ROS viz. super oxide radical, hydrogen peroxide and hydroxyl radical, is proposed to be the principle mechanism in As-intoxication [28]. ROS is not only responsible to induce peroxidative alteration of cellular lipids, carbonylation of proteins, fragmentation of DNA, reduction of cellular antioxidant molecules in the biological systems, but also affect the cell proliferation index by altering the signal transduction, cell cycle control, cellular differentiation and apoptosis [32]. The present study describes the protective role of AEIA against As toxicity employing suitable *in vitro* and *in vivo* preclinical assays. In this study, NaAsO_2_ (10 μM) exerted a significant reduction of the viability of murine hepatocytes *in vitro*. Hoechst staining of As-intoxicated hepatocytes confirmed the observation of cell viability assay and induction of apoptosis. Moreover, a distinct pattern of visible nuclei with chromatin condensation were noticed in Hoechst staining of NaAsO_2_-intoxicated hepatocytes. However, simultaneous incubation of hepatocytes with AEIA (400 μM) + NaAsO_2_ (10 μM) could reinstate the viability of cells, apparent from both cell viability and image assay.

Haematological and serum biochemical parameters stay earlier indicators of any pathophysiological state. In this study, a considerable reduction in total erythrocyte count and haemoglobin content was observed in NaAsO_2_-intoxicated mice, which is in accordance to the observation by Kavita et al. [33]. Cell viability and membrane integrity are directly related to each other. The increased concentrations of tissue specific enzymes in serum are the indications of membrane leakage. LDH and CK are the intracellular enzymes having high tissue specificity [31,34]. Increased levels of serum LAD and CK in NaAsO_2_ (10 μM) intoxicated mice supported the membrane damage and concomitant reduction of cell viability. As undergoes biotransformation in the liver where it binds with the thiol group of proteins and enzymes, thereby, interferes the integrity of plasma membrane of hepatocytes leading to leakage of AST and ALT in serum [34,35]. As-intoxication significantly increased serum level of urea, which may be indicative of protein catabolism and renal dysfunction [31]. In this study, NaAsO_2_-intoxicated mice exhibited hyperlipidemia evidenced by significantly higher cholesterol and triglycerides levels in serum, which is in accordance to the observation by Das et al. [1]. Increased synthesis or decreased removal of lipoproteins can be the possible reason of As-mediated hyperlipidemia. Concurrent treatment with AEIA along with NaAsO_2_ could significantly reinstate the haematological and serum biochemical parameters to near-normal status, which is an indicative of therapeutic potential of AEIA against As-intoxication.

NaAsO_2_-induced toxicity is associated with ROS generation which exerts direct toxic effect on critical tissues and attributes in apoptotic cell death. In this study, a significant increase in the accumulation of intercellular ROS was observed in both *in vitro* and *in vivo* assays. The inexorable generation of ROS during As-intoxication could be associated with elevated levels of lipid peroxidation and protein carbonylation. Both are principally involved in oxidative modifications of structural lipids and proteins of cell membrane leading to cell membrane damage [36]. However, co-administration of AEIA along with NaAsO_2_ could significantly inhibit lipid peroxidation and protein carbonylation via direct scavenging/quenching free radicals (ROS) produced during As-intoxication.

Ubiquinols (co-enzymes Q) functions as cellular electron transporters, thereby, act as antioxidants through scavenging ROS [37]. Co-enzymes Q are distributed within all major organales of cells including mitochondria, Golgi apparatus, endoplasmic reticulum, lysosomes, peroxysomes etc. A significant reduction of co-enzymes Q (Q9 and Q10) levels indicated the redox stress during As-intoxication. The accumulation of intracellular As may interfere with the activity of the enzyme viz. cytosolic NADPH-CoQ reductase accountable for the reduction of ubiquinone to ubiquinols [38]. Simultaneous administration of AEIA along with NaAsO_2_ could significantly reinstate co-enzymes Q levels in the tissues. The extracts might exert this effect through promoting clearance of As from the system.

The intra-cellular antioxidant system comprises antioxidant enzymes along with some non-enzymatic antioxidants. SOD and catalase are important radical scavenging enzymes. SOD acts by quenching the superoxide radicals leading to the formation of hydrogen peroxide and molecular oxygen, while, CAT is well known to detoxify hydrogen peroxide [39]. GPx coupled with GR accelerates reduction of lipid peroxide by the reaction with GSH [21]. GST protects cells against oxidants by removing free radicals. Significant reduction of these antioxidant enzymes during As-intoxication may be due to an enhanced production of ROS and/or down-regulation in the synthesis of antioxidant enzymes by persistent toxic effect of NaAsO_2_ [34]. Thiol-based antioxidant system, GSH, helps to protect cells from ROS [3]. The metabolism and excretion of As is dependent on GSH. In liver, As is metabolized into soluble mono-, di- or tri hydroxyl arsenic acid in presence of GSH [34]. As has strong affinity toward thiol group resulting depletion of GSH level in the tissues of NaAsO_2_-intoxicated mice confirmed this fact. Significant reduction of GR and GPx levels may be accountable to reduction of GSH in the tissue of As-intoxicated mice.

Intracellular As accumulation is thought to be major concern in chronic arsenicosis. The cellular uptake of arsenic has been investigated. In this study, NaAsO_2_-intoxication caused significantly high levels of bioaccumulation of As within the tissues. Increment of As burden within the tissues directly proportional to the redox stress via excessive generation of ROS. ROS down-regulates GSH and glutathione dependent enzymes which participate in As clearance via transforming into soluble methylated arsenic acid [40]. However, concurrent treatment with AEIA along with NaAsO_2_ could significantly reduce As accumulation within the tissues. The AEIA could exert this effect either by inhibiting ROS and restoring glutathione system to promote As clearance via bio-transformtion into soluble methylated arsenic acid and/or by promoting the clearance of intercellular As via chemo-transformation into soluble chelate with As. Presence of substantial quantities of dietary antioxidants (flavonoids, phenolics and ascorbic acid) and metal chelating metabolites (flavonoids and saponins) within the test extract would be accountable for As-clearance [36,41,42].

Intercellular redox stress plays an important role in DNA damage. The most reactive hydroxyl radical reacts with pyrimidine bases principally thiamine and remove a hydrogen atom from methyl group and -C-H bond of deoxyribose [43]. As a result, DNA strands are broken and/or DNAs are cross-linked. The DNA fragmentation plays a pivotal role in cell-death process in diabetic pathophysiology. In this study, significant DNA fragmentation was notices in the tissues of NaAsO_2_ intoxicated mice. However, AEIA could significantly counteract with DNA fragmentation probably by scavenging ROS and reducing oxidative stress.

Apoptosis, a self-destructive mechanism, is accomplished by mitochondrion-dependent (intrinsic) and/or independent (extrinsic) pathways. Redox stress attributes a principle role in initiation and execution of apoptotic pathway. A significant increase in the intracellular ATP concentration supported the incidence of apoptosis. Apoptosis is primarily executed by up-regulation of some pro-apoptotic and down-regulation of anti-apoptotic transcription proteins. Bcl 2, a member of anti-apoptotic protein, acts on the mitochondria to regulate the release of cytochrome c and initiate the caspases dependent apoptotic pathway. Bad, a pro-apoptotic protein, can modulate the pro-apoptotic processes by inhibiting Bcl 2 proteins [44]. Cyt C promotes ATP dependant formation of apoptosome by the aggregation of caspase 9 together with Apaf1 resulting in caspase 9 activation [45]. Caspase 9 then activates the rest of the caspase cascade by triggering the caspase 3 leading to apoptosis. The death receptor, Fas, Bid and caspase 8 comprise the extrinsic receptor mediated pathway. The Fas system activates caspase 8 which interacts with the intrinsic apoptotic pathway through activation of Bid, a pro-apoptotic protein [21]. In the present study, immunoblot analyses confirmed the involvement of both intrinsic and extrinsic pathways of apoptotic cell death in NaAsO_2_-intoxicated hepatocytes. However, AEIA could significantly reinstate these changes in transcription levels of apoptotic proteins near to normal status. Radical scavenging activity of AEIA would be accountable to anti-apoptotic activity of extract.

Finally, histological assessments showed that NaAsO_2_ caused abnormal histological changes within the tissues. However, pre-treatment with the extract could attenuate these histological changes and restore cyto-architecture near to the normal status.

In this study, we observed presence of phenolics (gallic acid and chlorogenic acid) and flavonoids (myricetin, quercetin and apigenin) in AEIA. Phenolics and flavonoids are potent antioxidants [46], which prevent apoptosis caused by oxidants [47]. Phenolics have been reported to exhibit anti-apoptotic potential through a Bcl-2 independent mechanism [48,49], while, anti-apoptotic activity of flavonoids is thought to be mediated at the mitochondria (intrinsic) levels [46]. Earlier investigation revealed the prophylactic effect of ascorbic acid against As-toxicity through antioxidant mechanism [50]. Presence of substantial quantity of ascorbic acid in AEIA would play crucial role in overall protective effect against As-toxicity. On other hand, phenolics and saponins have been reported to exhibit chelating properties to mop up arsenites from the body [42,51]. Therefore, phenolics, flavonoids, ascorbic acid and saponins within AEIA would offer a cumulative effect to ensure the protection against As-intoxication.

## Conclusion

**Figure 9:**
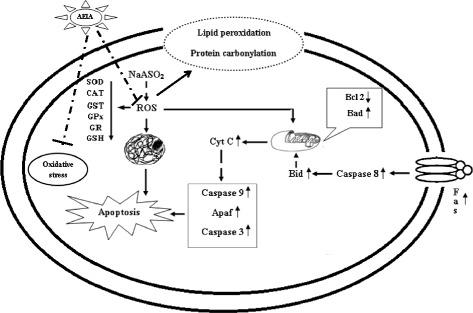
**Schematic presentation of probable protective mechanism (dotted lines) of AEIA against arsenicosis.**

In present study, it was found that aqueous extracts of the edible aerial parts of *I. aquatica* could attenuate toxic manifestation caused by NaAsO_2_. The mechanisms of arsenicosis and the overall protective role of aforementioned test material were confirmed in this study (Figure 9). Experimental data revealed that As-mediated generation of ROS principally contribute in the toxicosis of As. The results suggested that the tested extract could offer protection against NaAsO_2_-induced toxicity by counteracting oxidative stress and associated toxic manifestations and promoting As clearance from the tissues via metal chelating. Presence of dietary antioxidants namely flavonoids, phenolics and ascorbic acids in the test extracts could contribute in overall protection against arsenicosis. In conclusion, augmentation of *I. aquatica* can be a novel strategy for designing arsenicosis intervention in future.

## Additional file

Additional file 1:
**Additional file consists of supportive data on phytochemical investigation of aqueous extract of** ***I. aquatica.*** Figure S1a depicted the HPLC chromatograms of standard flavonoid markers viz. myricetin (R_t_: 4.1), quercetin (R_t_: 5.7) and apigenin (R_t_: 8.2) and flavonoids present within the test extract. Figure S1b showed HPLC chromatograms of standard phenolic markers viz. gallic acid (R_t_: 4.0) and chlorogenic acid (R_t_: 7.2) and phenolic compounds present within the test extract. Figure S1c showed HPLC chromatograms of standard ascorbic acid (R_t_: 3.9) and ascorbic acid present within the test extract.
