# Supplementary figures and images for: Ameliorative effect of water spinach, Ipomea aquatica (Convolvulaceae), against experimentally induced arsenic toxicity
Source: J Transl Med. 2015 Mar 5;13:81. doi: 10.1186/s12967-015-0430-3 (PMC4359489; doi:10.1186/s12967-015-0430-3)

## Slide 1
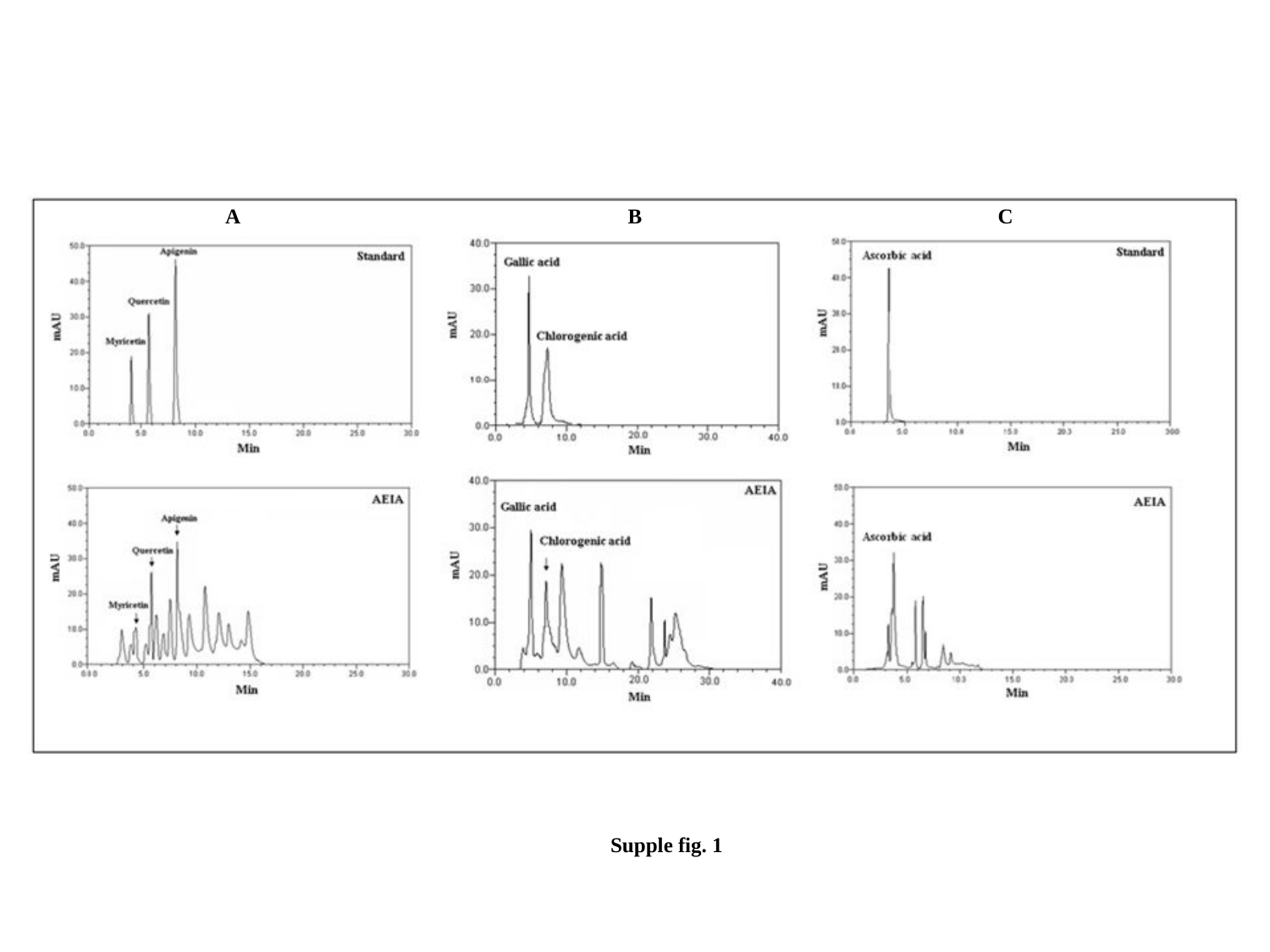

A
B
C
Supple fig. 1

Supplement: Additional file 1: — Additional file consists of supportive data on phytochemical investigation of aqueous extract of I. aquatica. Figure S1a depicted the HPLC chromatograms of standard flavonoid markers viz. myricetin (Rt: 4.1), quercetin (Rt: 5.7) and apigenin (Rt: 8.2) and flavonoids present within the test extract. Figure S1b showed HPLC chromatograms of standard phenolic markers viz. gallic acid (Rt: 4.0) and chlorogenic acid (Rt: 7.2) and phenolic compounds present within the test extract. Figure S1c showed HPLC chromatograms of standard ascorbic acid (Rt: 3.9) and ascorbic acid present within the test extract. [file 12967_2015_430_MOESM1_ESM.pptx]
